# Accumulation of mutations in nsp4, E, and the S2 subunit underlies mammalian cell tropism expansion and virulence attenuation of avian coronavirus

**DOI:** 10.1371/journal.ppat.1014147

**Published:** 2026-04-09

**Authors:** Yingfei Li, Xuehui Zhang, Rong Liang, Ruotong Li, Ruihua Yang, Liwei Zhang, Ye Zhao, Guozhong Zhang, Jing Zhao

**Affiliations:** 1 State Key Laboratory of Veterinary Public Health and Safety, College of Veterinary Medicine, China Agricultural University, Beijing China; 2 Key Laboratory of Animal Epidemiology of the Ministry of Agriculture, College of Veterinary Medicine, China Agricultural University, Beijing China; Chinese University of Hong Kong, HONG KONG

## Abstract

Infectious bronchitis virus (IBV), a Gammacoronavirus with strict host tropism, infects chickens and avian-derived primary cells but not mammalian cells. The classical Beaudette strain, isolated in the 1930s, is a well-established IBV strain capable of replicating in Vero cells. Although sporadic reports have described similar adaptation in other strains, the molecular mechanisms underlying IBV cell tropism remain unclear. Here, a prevalent IBV strain was passaged in embryonated eggs and primary host cells to generate quasispecies diversity, followed by an alternating host–nonhost passage strategy that enabled efficient replication in BHK-21 cells within ten passages. Reverse genetics identified mutations in nsp4 (T53I), the E protein (E10G), and the S2 subunit as key contributors to mammalian cell tropism expansion. Notably, some of these mutations were detectable prior to BHK-21 passaging, suggesting that pre-existing variants laid the foundation for subsequent adaptation. Furthermore, introducing this minimal mutation set into the GI-1 H120 virus backbone similarly enabled efficient replication in BHK-21 cells, yielding titers of 10^5.4^ TCID_50_/ml. In vivo, the nsp4–E–S2 mutant exhibited a 40% reduction in mortality compared with the wild type, and RNA sequencing revealed attenuated inflammatory responses. Collectively, our findings indicate that mammalian cell tropism expansion in IBV is associated with the stepwise selection of pre-existing variants, resulting in coordinated mutations in the S protein and other structural and non-structural proteins that reduced pathogenicity while maintaining protective efficacy, highlighting BHK-21 cells as a promising platform for next-generation vaccine development.

## Author summary

Infectious bronchitis virus (IBV), a member of the γ-coronavirus genus, is primarily controlled via live attenuated vaccines. However, the production of these vaccines depends on embryonated chicken eggs, which are plagued by supply constraints, inherent risks of adventitious agent contamination, and potential interference from egg-derived components on immunogenicity. While cell-based manufacturing offers distinct advantages, the scarcity of permissive cell lines and the incomplete understanding of the molecular mechanisms underlying IBV cell tropism have long hindered its practical implementation. Utilizing a circulating GI-19 strain, we show that IBV adaptation to mammalian cells requires coordinated mutations not only in the spike (S) protein but also in non-structural protein 4 (nsp4) and the envelope (E) protein. Collectively, these mutations enable tropism expansion while concomitantly attenuating viral virulence. These molecular determinants were further validated using the GI-1 H120 virus backbone. This study provides critical mechanistic insights that are essential for the rational development of cell culture-derived live IBV vaccines.

## Introduction

Avian infectious bronchitis virus (IBV) is an enveloped, positive-sense single-stranded RNA virus belonging to the genus *Gammacoronavirus* within the family *Coronaviridae* [1]. The IBV genome is approximately 28 kb in length and encodes at least 11 open reading frames (ORFs) [2,3]. The two overlapping ORFs, ORF1a and ORF1b, occupy the first two-thirds of the genome and are translated into large polyproteins, which are subsequently cleaved into 15 nonstructural proteins. Coronavirus transcription proceeds via a unique discontinuous mechanism, in which the leader transcription regulatory sequence (TRS-L) at the 5′ end switches templates with body TRS (TRS-B) sites upstream of each gene, generating a nested set of subgenomic RNAs (sgRNAs) [4]. These sgRNAs are translated into the four structural proteins—spike (S), envelope (E), membrane (M), and nucleocapsid (N)—as well as accessory proteins including 3a, 3b, 4b, 5a, and 5b [5]. Since its first description in the 1930s, IBV has been recognized as one of the most economically devastating respiratory pathogens, causing highly contagious disease in chickens and posing a major threat to the global poultry industry [1,6]. The virus primarily replicates in the epithelial cells of the respiratory tract, because of the lack of a proofreading mechanism in the viral RNA-dependent RNA polymerase, mutations accumulate rapidly during replication [7,8]. The unique discontinuous transcription mechanism of coronaviruses facilitates the generation of recombinant viruses through random template switching of the polymerase [4]. The widespread application of IBV vaccines has further imposed strong immune selection pressure on circulating strains [9], thereby accelerating viral evolution and contributing to the continuous emergence of novel IBV variants [10,11]. Some of these variants display expanded tissue tropism and are capable of invading multiple organs, leading to renal damage, impaired oviduct development, reduced egg production, and increased mortality in young chickens [12]. Among these, the GI-19 lineage, also known as the QX-type, has become the most prevalent genotype worldwide [13].

An intranasal live-attenuated vaccine can effectively control respiratory virus infections by mimicking natural infection, expressing multiple viral antigens, and inducing both mucosal and systemic immune responses [14]. In recent years, the use of suspension cell lines for the production of viral vectors and vaccines has received increasing attention. Several mammalian cell substrates have already been licensed for vaccine production, such as Madin–Darby canine kidney (MDCK) cells for inactivated influenza vaccines [15], Vero cells for inactivated COVID-19 vaccines [16], and human embryonic kidney (HEK) 293 cells for adenovirus-vectored SARS-CoV-2 vaccines [17]. Meanwhile, other cell lines, including baby hamster kidney (BHK-21) cells for foot-and-mouth disease virus (FMDV) or Zika virus and Marc-145 cells for porcine reproductive and respiratory syndrome virus (PRRSV), are being actively investigated as potential platforms for vaccine development [18]. Whereas, field isolates of IBV exhibit strict host specificity and can only infect chickens [19]. To date, even for avian-origin cells, viral replication is limited to primary chicken embryo kidney (CEK) cells, making it difficult to realize the goal of cell culture-based vaccine production.

Viral infection is initiated by the attachment of virions to specific receptors on the host cell surface, making receptor recognition a critical determinant of cellular and tissue tropism [20]. In coronaviruses, this process is mediated by the envelope-anchored S glycoprotein. Mutations in the receptor-binding domain (RBD) of SARS-CoV-2 have been shown to strengthen viral infectivity in cells expressing ACE2 orthologs from non-human animals, particularly those that were less susceptible to the ancestral strain [21]. These changes also enhanced cross-species infection potential in animal models such as mice and ferrets, and were found to correlate with the cumulative prevalence of RBD mutations in humans [22,23]. Similarly, the cellular adaptability of porcine epidemic diarrhea virus (PEDV) depends largely on the S1 subunit and the N-terminal region of S2 [24,25]. Collectively, these observations indicate that spike protein mutations play an indispensable role in cross-species transmission and the expansion of host and cell tropism among coronaviruses. Interestingly, the molecular mechanism of IBV cell tropism expansion remains largely unknown, among all IBV strains, the Beaudette strain — after extensive serial passaging in chicken embryos and about 65 passages in Vero cells [26,27] — has shown efficient adaptation to mammalian cell lines. Similarly, the commercial vaccine strain H120 was reported to acquire the ability to efficiently replicate in Vero cells after about 80 serial passages [28]. Both Beaudette and H120 belong to the GI-1 genotype, which is genetically distinct from the currently predominant GI-19 (QX-type) lineage. Due to the marked genetic divergence between these genotypes, amino acid sequence comparison alone provides limited insight into the molecular basis underlying cell adaptation. Previous studies have suggested that the S2 subunit of the Beaudette spike protein plays a critical role in its adaptation to Vero cells [29] and intestinal tropism [30]. Yet fundamental questions remain unanswered: What specific role does the spike protein play in mammalian cell infection? And could additional viral proteins contribute to this adaptation process?

In this study, we aimed to investigate the molecular mechanisms of IBV cell tropism. Considering that serial passaging of IBV in Vero cells leads to the emergence of an additional furin cleavage site at S2′—a feature known to enhance tissue tropism and virulence [31,32]. BHK-21 cells are commonly used for the rescue of various RNA viruses and serve as a key platform for the propagation and production of viruses such as foot-and-mouth disease virus (FMDV) and Zika virus [33,34]. Building on this, we achieved adaptation of IBV to BHK-21 cells by alternately passaging the virus between host cells and the mammalian cells line. Using reverse genetics, we demonstrated that while mutations in the S2 subunit of the spike protein are necessary, they are not sufficient to confer replication competence in mammalian cells. Additional cooperative mutations in nsp4 (T53I) and the E (E10G) protein are required for IBV replication in BHK-21 cells. Notably, the adaptive mutations vary across different cell types; however, the key mutation set identified in this study was sufficient to confer BHK-21 cell tropism on the H120 strain, while simultaneously attenuating its virulence in one-day-old chickens. Moreover, the adapted virus conferred effective protection against subsequent WT challenge. From a vaccine production perspective, this discovery offers a promising strategy to break through the limitations of traditional embryonated egg–based systems, providing a scientific foundation and practical guidance for the development of safe, high-yield, cell culture–based live vaccines for IBV.

## Results

### Adaptation of IBV to mammalian cells

**Fig 1:**
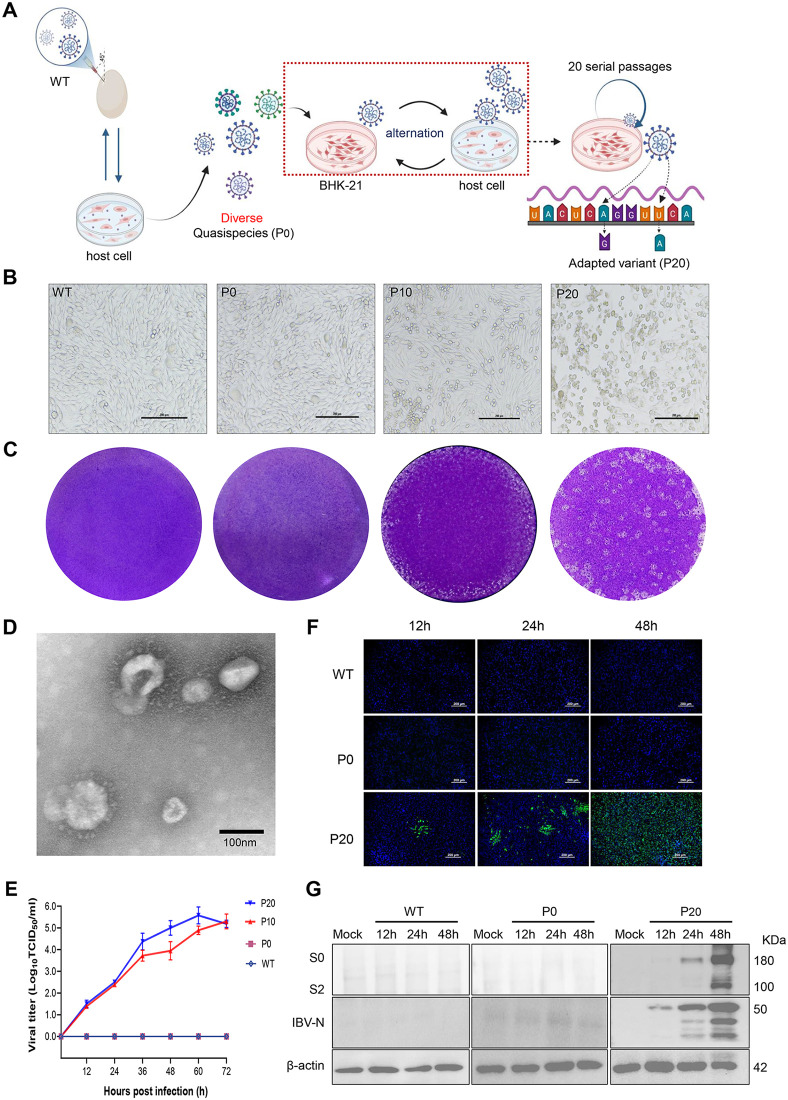
Adaptation of IBV to mammalian cells. **(A)** Schematic diagram of the alternating passage procedure. WT, wild type; host cell, chicken embryo kidney (CEK) cells. This image was created with https://BioRender.com/7nsgzs1. **(B)** Cytopathic effects (CPE) observed in infected cells. **(C)** Plaque morphology of the adapted virus. **(D)** TEM images of purified P_20_ virions. Scale bar: 100 nm. **(E)** Viral replication kinetics in BHK-21 cells. **(F)** Immunofluorescence detection of IBV S2 protein in infected cells. BHK-21 cells were infected with the wild-type (WT), P_0_, or mammalian-adapted (P_20_) strains of IBV. Cells were fixed at 12-, 24-, and 48 hpi. Scale bar: 200 μm. **(G)**Western blot analysis showing the expression of IBV S2 and N proteins in cells infected with the WT, P_0_, and adapted P_20_ strains at 12-, 24-, and 48 hpi.

Despite prior attempts to introduce either the full-length spike gene of the Vero-adapted Beaudette-P65 strain or its S2′ furin cleavage motif (RRKR) into the prevalent QX-type (GI-19) IBV strain, no adaptation to Vero cells was observed. We also observed that IBV generally loses infectivity after 1 ~ 2 passages in non-host cells. Based on this, we employed an alternating passage strategy between natural host cells and non-host cells (Fig 1A): wild-type IBV was first serially passaged in embryonated chicken eggs and primary CEK cells to generate a diverse viral quasispecies population (P0). The viral quasispecies was then inoculated into BHK-21 cells, followed by alternating passages between BHK-21 and CEK cells, in this strategy, CEK cells served to amplify the limited number of viral particles that were able to adsorb to BHK-21 cells, thereby preventing loss of the virus during passages in non-host cells. At passage 10 (P10), BHK-21 cells began to exhibit rounding and shrinkage, and by P20, the cytopathic effect became more pronounced, with cell detachment (Fig 1B). Plaque assays showed that plaques at P20 were larger and more distinct than those at P10, whereas the wild-type virus did not form plaques (Fig 1C). Transmission electron microscopy (TEM) revealed virus particles exhibiting characteristic coronavirus-like morphology (Fig 1D). At P_20_, the virus reached its replication peak at 60 hours post-infection, with a titer of 10^5.57^ TCID_50_/ml, representing an increase compared with P_10_. In contrast, no detectable viral titers were observed in the wild-type strain or in the pre-adaptation quasispecies population (P0) (Fig 1E). Indirect immunofluorescence assays (Fig 1F) and Western blot analysis (Fig 1G), using monoclonal antibodies against both the S2 and N proteins, showed that viral protein expression progressively increased over time for P20, whereas no detectable viral protein expression was observed for WT or P0. Simultaneously, viral titers were quantified and endpoint titers were determined in CEK cells. The results were consistent, showing that although P0 exhibited detectable titers, there was no increasing trend over time (S1A Fig). When evaluated in CEK cells, P0, P10, and P20 demonstrated significantly higher replication titers compared with WT. (S1B Fig). Similarly, endpoint titers assessed by genomic copy number showed gradual accumulation of viral genomes over time for P10 and P20, whereas WT remained unchanged. P0 displayed minor fluctuations, but no significant increase was observed (S1C Fig). Collectively, these results demonstrate that the GI-19 genotype field strain can rapidly acquire BHK-21 cell tropism through serial alternating passages in non-permissive cells and host cells.

Subsequently, we evaluated the general applicability of this alternating-passaging strategy. When the wild-type was serially passaged directly in DF1 cells (avian-derived) or alternately between DF1 and its natural host cells, no adaptation was achieved. In contrast, the BHK-21–adapted IBV, although unable to directly replicate in DF1 cells, successfully acquired replication competence after alternating passages (S1D Fig), reaching a titer of 10^4.3^ TCID_50_/ml (S1E Fig). The BHK-adapted virus was further inoculated into mammalian cell lines, including 293T, A549, and Vero-E6 cells (S1F Fig). No detectable viral amplification was observed. These findings indicate that the amino acid determinants required for adaptation differ among cell types, and that prior adaptation to BHK-21 cells facilitates the subsequent expansion of IBV tropism to DF1 cells. Moreover, the presence of a diverse quasispecies population remains a prerequisite for such adaptive evolution.

### The BHK-adapted virus harbors multiple mutations

**Fig 2:**
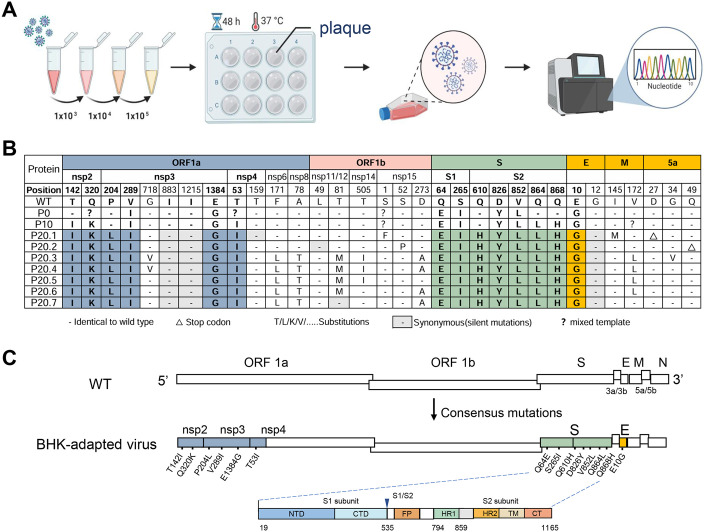
Whole-genome mutation analysis of IBV adaptation to BHK-21 cells. **(A)** Schematic diagram of plaque purification and sequencing workflow. This image was created with https://BioRender.com/e7rno2s. **(B)** Genome-wide mutation mapping, with P_20_._1_–P_20.7_ representing seven randomly selected plaques. **(C)** Fourteen consensus mutations identified across all clones.

Direct sequencing of the BHK-adapted virus revealed numerous double peaks, indicative of the presence of diverse quasispecies. The high number of mutations made it difficult to determine which changes were most closely associated with BHK-21 cell adaptation. To address this, P_20_ virus was used to infect cells, followed by three rounds of plaque purification (Fig 2A) Seven well-defined plaques were randomly selected, and each was subjected to genome-wide PCR amplification and Sanger sequencing. Comparison from WT to P_0_ indicated that many mutations had already accumulated during passages in embryonated chicken eggs and CEK cells. The seven plaques carried distinct sets of mutations distributed across nonstructural and structural proteins (except for the N protein). Notably, mutations in the accessory 5a gene introduced a premature stop codon, leading to early termination of translation (Fig 2B). Analysis of seven plaques identified 14 consensus mutations, distributed across the viral genome as follows: nsp2 (2 mutations), nsp3 (3 mutations), nsp4 (1 mutation), S gene (7 mutations), and E gene (1 mutation). (Fig 2C). Overall, these findings suggest that BHK-21 cell adaptation of IBV is driven by a complex mutational landscape, with predominant changes localized to the spike protein, especially the S2 subunit.

### S protein is necessary but not sufficient for IBV cell adaptation, requiring cooperation with Nsp4 (T53I) and E (E10G)

**Fig 3:**
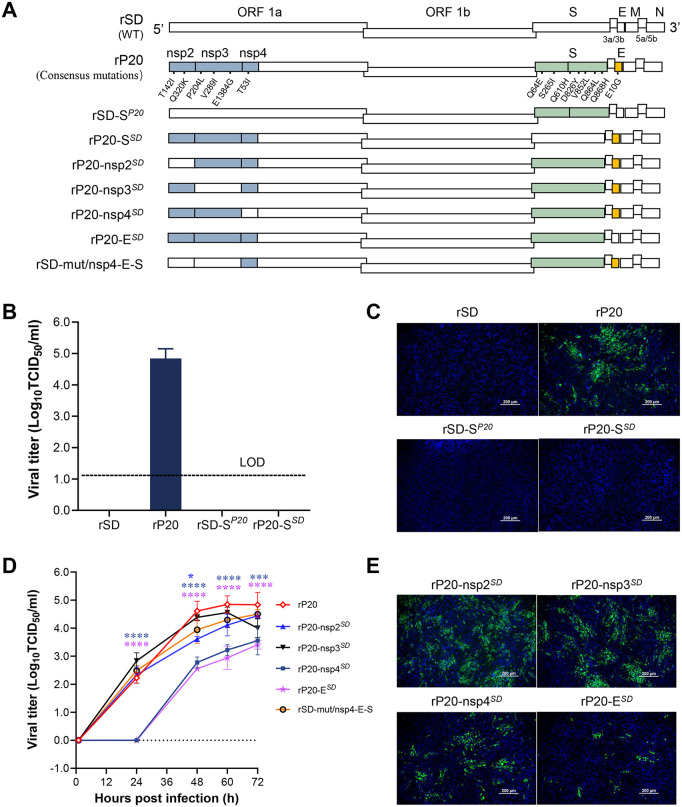
Roles of structural and nonstructural proteins other than the S protein in IBV cell tropism. **(A)** Schematic representation of recombinant viruses. **(B)** Viral titers of the recombinant viruses with S protein replacement. LOD, limit of detection. **(C)** Indirect immunofluorescence assay of cells infected with S protein–replaced viruses, images taken at 72 hpi. **(D)** Viral titers of recombinant viruses in which nsp2, nsp3, nsp4, or E of rP_20_ were individually reverted to the WT sequence. **(E)** Indirect immunofluorescence assay of cells infected with rP_20_ derivatives in which nsp2, nsp3, nsp4, or E were reverted to WT, images taken at 72 hpi.

Unlike previous work that predominantly centered on the spike protein, we first highlighted the involvement of other structural and nonstructural proteins in the process of IBV cell tropism. The schematic representation of the substitutions is indicated using different colors (Fig 3A). The rescued virus harboring all 14 consensus mutations was designated rP_20_, while the wild-type rescued strain was referred to as rSD. To dissect the contribution of the spike protein and non-spike regions, reciprocal chimeric viruses were constructed: the S gene of rP20 was introduced into the rSD backbone, resulting in the recombinant rSD-S^P20^, and conversely, the S gene of rSD was inserted into the rP20 backbone, generating rP20-S^SD^. Viral titration assays showed that only rP20 replicated efficiently in BHK-21 cells, reaching a titer of 10^4.8^ TCID_50_/ml. Neither the S gene substitution alone (rSD-S^P20^) nor the non-S mutations retained in the rP20 backbone (rP20-S^SD^) were sufficient to confer BHK-21 cell adaptation (Fig 3B). Consistent with these findings, IFA detected fluorescence signals exclusively in rP20-infected cells (Fig 3C). These results demonstrate that the S protein alone cannot drive the adaptation of IBV from avian to mammalian cells.

To further investigate the contribution of the mutations—nsp2 (T142I, Q320K), nsp3 (P204L, V289I, E1384G), nsp4 (T53I), and E (E10G)—to BHK-21 cell adaptation, each mutation was individually reverted to its corresponding wild-type residue. Reversion of nsp4 at position 53 from I to the wild-type T (rP20-nsp4^*SD*^) led to an approximately 40-fold reduction in viral titer at 60 dpi, whereas reversion of E at position 10 from G to the wild-type E (rP20-E^*SD*^) caused a ~ 79-fold decrease. In contrast, reverting the nsp2 (I142T, K320Q) and nsp3 (L204P, I289V, G1384E) mutations did not alter the viral titer of rP_20_, furthermore, the rSD backbone simultaneously harbored the nsp4 (T53I), E (E10G), and S mutations (rSD-mut/nsp4-E-S), its replication efficiency in BHK-21 cells was restored to a level comparable to that of rP20 (Fig 3D). Consistently, immunofluorescence assays revealed markedly reduced fluorescence intensity for both rP20-nsp^*SD*^ and rP20-E^*SD*^ (Fig 3E), highlighting the critical roles of the nsp4 (T53I) and E (E10G) mutations in facilitating IBV adaptation to mammalian cell lines.

### The S2 subunit determines IBV cell tropism in the context of nsp4 (T53I) and E (E10G) mutations

**Fig 4:**
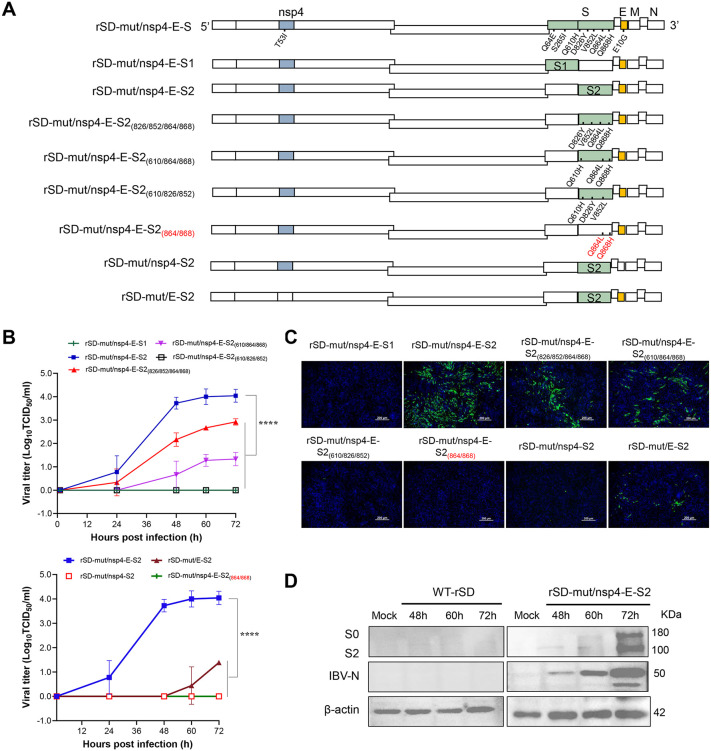
The nsp4(T53I), E(E10G), and S2 subunit collectively determine IBV cell tropism. **(A)** Schematic representation of recombinant viruses. **(B)** Replication kinetics of recombinant IBV constructs used to dissect the contribution of individual spike subunits. S1 or S2 was replaced while retaining the nsp4 (T53I) and E (E10G) mutations, followed by further reduction of mutation combinations to identify the minimal set of determinants required for BHK-21 cell adaptation. ****: p < 0.0001. **(C)** Indirect immunofluorescence assay showing the combined effect of nsp4(T53I), E(E10G), and the S2 subunit, images taken at 72 hpi. **(D)** Western blot analysis of protein expression in BHK-21 cells infected with WT and rSD-mut/nsp4-E-S2 (carrying 7 mutations in total) at 48-, 60-, and 72 hours post-infection.

Reversion of nsp4 at position 53 and E protein at position 10 to their wild-type residues markedly reduced the replication titer of rP20 in BHK-21 cells. Therefore, the adaptive mutations nsp4 (T53I) and E (E10G) were retained to further determine whether the S1 or S2 subunit of the spike protein governs IBV cell tropism (Fig 4A). Using rSD as the backbone, introduction of nsp4 (T53I), E (E10G), and five mutations located in the S2 subunit (rSD-mut/nsp4-E-S2) resulted in a viral titer of approximately 10^4^ TCID₅₀/ml. In contrast, simultaneous introduction of nsp4(T53I), E (E10G), and S1 subunit mutations (rSD-mut/nsp4-E-S1) yielded no detectable replication. Among the S2 mutations, Q610H emerged between P_10_ and P_20_, D826Y and V852L appeared during adaptation from WT to P_0_, Q864L and Q868H arose between P_0_ and P_10_. Reverting residue 610 in S2 to its wild-type sequence (rSD-mut/nsp4-E-S2(826/852/864/868)) or reverting residues 826 and 852 to wild-type (rSD-mut/nsp4-E-S2(610/864/868)) significantly reduced viral titers, whereas restoration of residues 864 and 868 to wild-type (rSD-mut/nsp4-E-S2(610/826/852)) completely abolished replication. These findings demonstrate that the adaptive mutations within the S2 subunit play a pivotal role in mediating IBV adaptation to mammalian cells. Both the combinations of E and S2 mutations (rSD-mut/E-S2), nsp4 and S2 mutations (rSD-mut/nsp4-S2), as well as the combination of nsp4, E, and S2_(864+868)_ mutations (rSD-mut/nsp4-E-S2(_864/868_)), resulted in markedly reduced or even undetectable replication compared with rSD-mut/nsp4-E-S2 (Fig 4B). Immunofluorescence assay further supported these findings (Fig 4C), and Western blot analysis confirmed that rSD-mut/nsp4-E-S2 was capable of sustained replication in BHK-21 (Fig 4D). These results demonstrate that the S2 subunit of IBV determines cell tropism, which depends on the presence of the nsp4 (T53I) and E (E10G) mutations.

### Minimal mutations were sufficient to confer mammalian cell tropism on GI-1 rH120 strain

**Fig 5:**
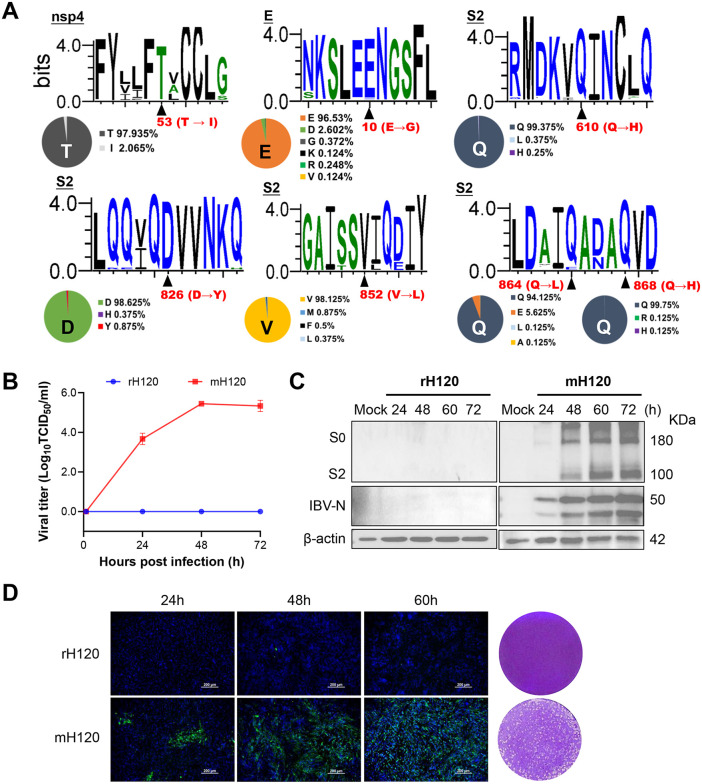
Effects of the adaptive mutations nsp4(T53I), E(E10G), and the S2 subunit on the rH120(GI-1). **(A)** Conservation analysis of the adaptive mutations based on 800 complete IBV genome sequences retrieved from NCBI. Highly conserved amino acid sites were defined as residues with ≥95% sequence identity. This image was created with WebLogo 3 - Create **(B)** Viral titers of the rescued rH120 and its mutant (mH120) in BHK-21 cells. **(C)** Western blot analysis of protein expression in BHK-21 cells infected with rH120 and mH120. **(D)** Left: Indirect immunofluorescence assay (IFA) detecting S2 protein expression of rH120 and mH120 in BHK-21 cells using an anti-IBV S2 monoclonal antibody. Right: Plaque morphology of rH120 and mH120.

To assess the conservation and potential universality of the adaptive mutations, a total of over 800 full-length IBV genome sequences representing different genotypes were downloaded from the NCBI database for comparative analysis. The analysis revealed that nsp4 (T53), residues in the S2 subunit (Q610, D826, V852, Q868), and E (E10) are highly conserved among IBV strains (≥95% sequence identity), except for Q864 in the S2 subunit, which exhibited a slightly lower conservation rate of 94.125% (Fig 5A). Accordingly, these adaptive mutations—nsp4 (T53I), E (E10G), and the five substitutions within the S2 subunit—were introduced into the genetic backbone of the commercially available GI-1 rH120 strain using reverse genetics. Remarkably, the recombinant H120 mutant (mH120) acquired the ability to replicate in BHK-21 mammalian cells, reaching titers of approximately 10^5.4^ TCID_50_/ml, whereas no detectable replication was observed for the rH120 strain (Fig 5B). Western blot analysis confirmed viral protein expression in mH120-infected BHK-21 cells, with peak levels detected at 48–60 hours post-infection, while no bands were observed for the rH120 strain (Fig 5C). Consistently, IFA revealed strong fluorescence signals and clear plaque formation in BHK-21 cells infected with the mutant virus (Fig 5D). The recombinant mH120 virus was serially passaged eight times in BHK-21 cells, and Sanger sequencing confirmed that all adaptive mutations were stably maintained without additional substitutions (S2 Fig). Taken together, our research demonstrates that the combination of nsp4 (T53I), E (E10G), and S2 subunit mutations not only enables the SD strain (GI-19 genotype) to adapt to mammalian cells but also allows the rH120 strain (GI-1 genotype) to rapidly acquire replication competence in heterologous cells.

### BHK adaptation enhances replication and cell–cell fusion in CEK cells without affecting early entry

**Fig 6:**
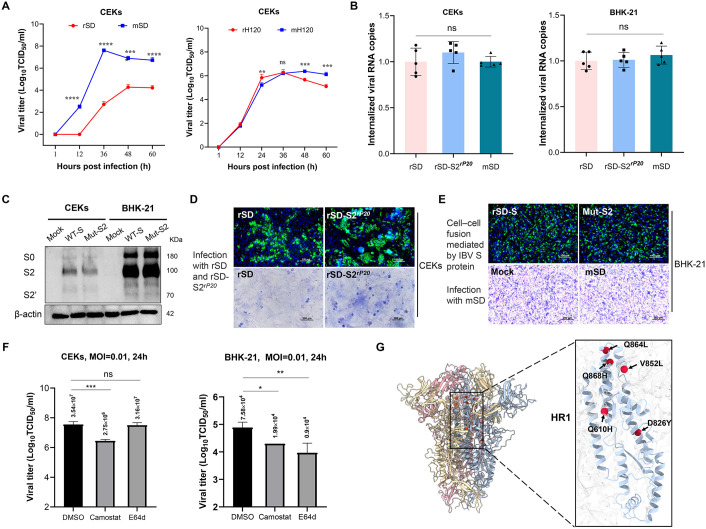
Functional effects of S2 mutations. **(A)** Replication kinetics of rSD and mSD, and rH120 and mH120 in CEK cells at an MOI of 0.01. Supernatants were collected at 1, 12, 36, 48, and 60 hpi for viral titration. **(B)** Total internalized viral genome copies of rSD, rSD-S2rP20, and mSD in CEK and BHK-21 cell. **(C)** Western blot analysis of WT-S and S2 mutant plasmids expressed in CEK and BHK-21 cells, showing S protein cleavage. **(D)** Syncytium formation in CEK cells induced by infection with rSD or rSD-S2rP20. **(E)** Syncytium formation in BHK-21 cells induced by plasmid-transfected S proteins or mSD infection. **(F)** Effects of Camostat (20 μM) or E64d (20 μM) treatment on mSD replication in CEK and BHK-21 cells. **(G)** Structural modeling of the S protein trimer using AlphaFold3, visualized with UCSF ChimeraX.

To evaluate the replication kinetics of BHK-adapted IBV in primary chicken cells, we compared the viral titers of rSD and mSD, as well as rH120 and mH120, in CEK cells. The results showed that the BHK-adapted mSD strain exhibited significantly higher titers than rSD, whereas no significant difference was observed in the peak replication levels between rH120 and mH120 (Fig 6A). To investigate the functional contribution of S2 mutations, we generated a recombinant virus, rSD-S2^rP20^, by introducing the S2 mutations into the rSD genomic backbone. This mutant virus displayed no significant differences in early entry steps, including attachment and internalization, in both CEK and BHK-21 cells (Fig 6B). Consistently, transfection of either the wild-type S protein (WT-S) or the S2 mutant into CEK and BHK-21 cells revealed comparable S2 and S2′ cleavage patterns by Western blot, indicating that the S2 mutations do not affect viral early entry or S protein cleavage (Fig 6C).

Notably, in CEK cells, the S2 mutant induced markedly enhanced cell–cell fusion compared with rSD (Fig 6D). In contrast, no syncytium formation was observed in BHK-21 cells, whether S protein was expressed via plasmid transfection or during mSD infection (Fig 6E). To determine whether the differences in syncytium formation were associated with distinct entry mechanisms, CEK and BHK-21 cells were treated with camostat mesylate, a serine protease inhibitor targeting transmembrane serine proteases (e.g., the TMPRSS family), or E64d, a cell-permeable cysteine protease inhibitor that blocks cathepsin activity. At 10 μM and 20 μM, neither compound affected cell viability (S3 Fig). In CEK cells, Camostat significantly inhibited viral replication, whereas in BHK-21 cells, E64d treatment substantially reduced viral titers (Fig 6F), indicating that IBV may utilize distinct endocytic entry pathways depending on the host cell type. Finally, structural modeling revealed that all identified S2 mutations are located near the HR1 domain, with Q864L and Q868H positioned adjacent to the HR1 core region (Fig 6G), further supporting that S2 mutations may enhance or alter membrane fusion activity by modulating HR1 conformation.

### IBV mutants exhibit reduced pathogenicity in chickens

**Fig 7:**
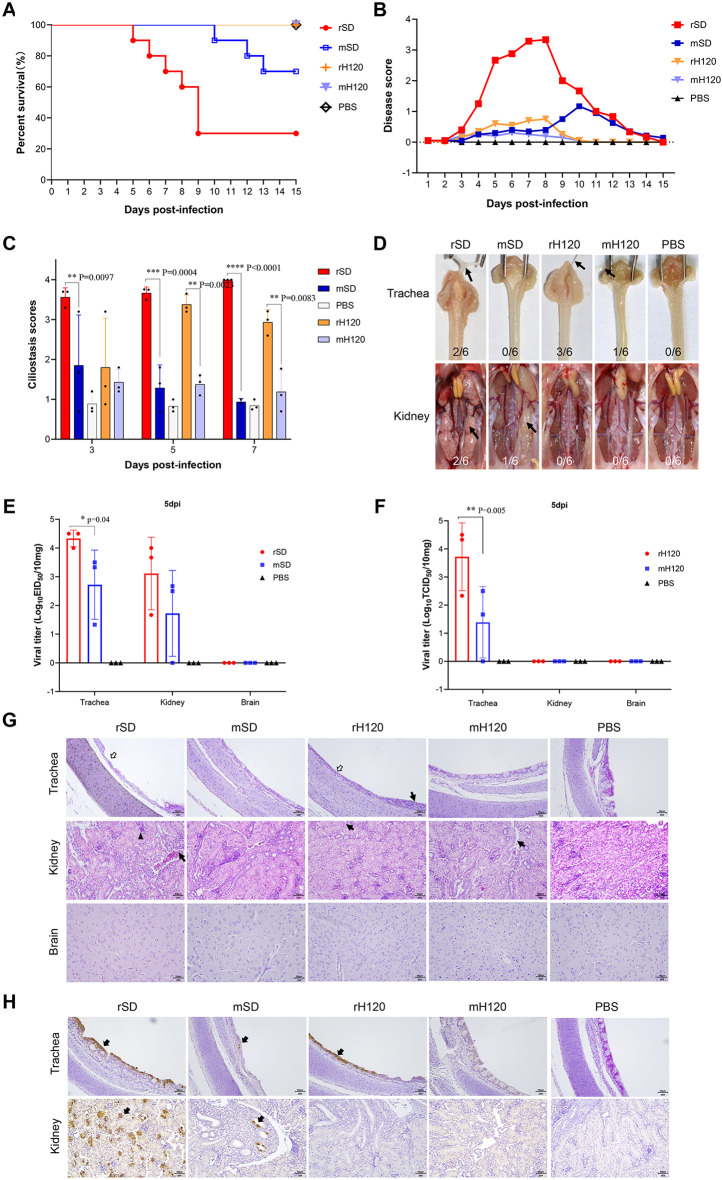
Infection of 1-day-old SPF chickens with rSD, rH120, and their mutants. **(A)** Survival curves (n = 10). **(B)** Clinical symptom scores. **(C)** Tracheal ciliary activity scores. **(D)** Gross pathological lesions in the trachea and kidneys. **(E)** Viral titers in the trachea, kidney, and brain at 5 dpi with rSD, mSD and PBS. **(F)** Viral titers in the trachea, kidney, and brain at 5 dpi with rH120, mH120 and PBS. **(G)** Histopathological changes in the trachea, kidneys and brain. Open arrows indicate ciliary epithelial shedding; thin arrows denote inflammatory cell infiltration; black arrowheads indicate necrosis and desquamation of renal tubular epithelial cells. Scale bar: 50 μm. **(H)** Immunohistochemical detection of IBV antigens in trachea and kidney tissues. Black arrows indicate positive signals. IBV-N monoclonal antibody was used as the primary antibody. Scale bar: 50 μm.

To assess whether the mutations in nsp4, E, and the S2 subunit altered the pathogenicity of IBV in its natural host, one-day-old SPF chickens were inoculated and monitored for two weeks. The wild-type SD strain exhibited high virulence with a mortality rate of 70%, whereas the mutant strain mSD caused only 30% mortality with delayed onset of death. Neither the rH120 strain nor its mutant mH120 induced any mortality (Fig 7A). Clinical observations showed that chickens inoculated with SD began to exhibit severe respiratory distress by 3 days post-infection (dpi), reaching peak morbidity at 8 dpi. In contrast, mSD-infected chickens displayed only mild clinical signs, with three of ten birds showing symptoms at 5 dpi that became more pronounced by 10 dpi. rH120-infected birds exhibited mild mouth breathing, while those in the mH120 group remained largely asymptomatic. All birds recovered by 14 dpi (Fig 7B). Because IBV primarily infects the upper respiratory tract, tracheal ciliary activity was used as a sensitive indicator of infection. Ciliary beating remained largely preserved in the mSD- and mH120-infected groups, whereas marked ciliary dysfunction and ciliostasis were evident in the rSD- and rH120-infected groups. (Fig 7C). Gross lesions at necropsy (5–7 dpi) were consistent with these findings. Two of six chickens in the rSD group showed tracheal mucus and hemorrhagic spots (2/6) and urate deposition in the kidneys (2/6). In contrast, mSD-infected chickens showed no tracheal lesions (0/6) and only one bird exhibited mild kidney swelling (1/6). In the rH120 group, three of six birds presented with tracheal mucus (3/6), whereas no lesions were detected in the mH120 group (0/6) (Fig 7D). In trachea, kidney, and brain tissues collected at 5 dpi, rSD exhibited significantly higher viral titers in the trachea compared with mSD, while no significant difference was observed in the kidneys, and no infectious virus was detected in the brain (Fig 7E). Similarly, in the rH120 group, viral titers in the trachea were significantly higher than those of mH120, kidney and brain tissues contained no detectable infectious virus (Fig 7F). Histopathological examination revealed severe epithelial desquamation and lamina propria exposure in tracheas of rSD- and rH120-infected chickens, accompanied by inflammatory infiltration in renal tubules in the rSD group. In contrast, mSD- and mH120-infected birds exhibited intact tracheal cilia and minimal infiltration of inflammatory cells in the kidneys, the brain tissues revealed no lesions in any group. (Fig 7G). Immunohistochemistry confirmed these findings: strong antigen signals were detected in trachea and kidney tissues of rSD-infected chickens, whereas signals were markedly weaker in mSD-infected birds. Similarly, rH120 produced stronger staining in tracheal tissues than mH120 (Fig 7H). In summary, IBV adapted to BHK-21 mammalian cells exhibits markedly reduced pathogenicity upon return to the chicken host, and shows no evidence of expanded tissue tropism.

### Differentially expressed genes (DEGs) in chickens infected with wild-type and mutant IBV by RNA sequencing (RNA-seq) analysis

**Fig 8:**
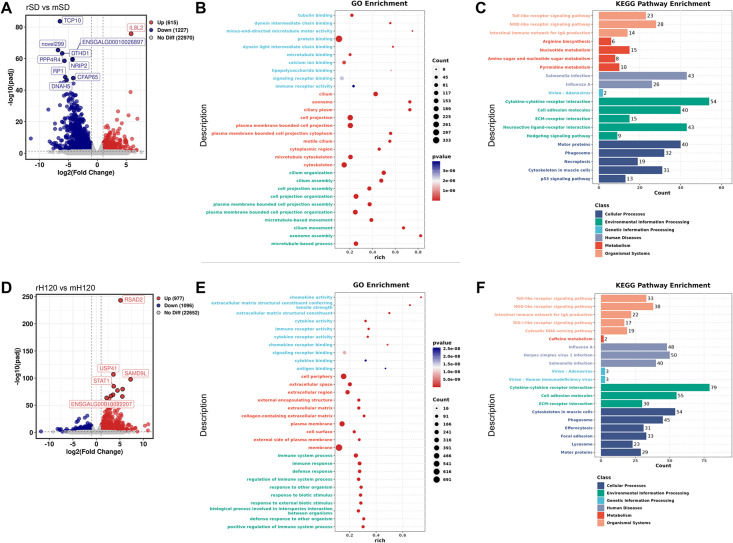
Differential expression profiling of tracheal tissues from chickens infected with wild-type and mutant IBV strains. **(A)** Volcano plot showing differentially expressed genes (DEGs) between rSD- and mSD-infected tracheal tissues at 5 days post infection. **(B)** Gene Ontology (GO) enrichment analysis of DEGs in the rSD VS mSD. **(C)** Kyoto Encyclopedia of Genes and Genomes (KEGG) pathway enrichment analysis for rSD VS mSD. **(D)** Volcano plot of DEGs identified between rH120- and mH120-infected tracheal tissues. **(E)** GO enrichment dot plot for DEGs in the rH120 VS mH120. **(F)** KEGG pathway enrichment bar plot for rH120 VS mH120.

To elucidate the mechanisms underlying the attenuated pathogenicity of the adapted strains, we performed RNA-seq analysis on tracheal tissues collected at 5 dpi from chickens infected with rSD, its adapted mutant mSD; rH120, or its adapted mutant mH120. Differentially expressed genes (DEGs) were identified for each comparison. Alignment to the reference genome yielded high mapping rates ranging from 90.47% to 97.05%. In the rSD versus mSD comparison, a total of 1,842 DEGs were detected. Compared with mSD, rSD infection predominantly upregulated the inflammatory cytokine IL8L2, while downregulating genes associated with apoptosis inhibition (TCP10), ciliary assembly (PPP4R4), cellular ion homeostasis (DTHD1), as well as several uncharacterized genes (Fig 8A). Gene Ontology (GO) enrichment analysis revealed that these DEGs were mainly enriched in cytoskeleton organization, microtubule dynamics, cilium structure, and ciliary movement (Fig 8B), consistent with the observation that the more virulent rSD strain causes irreversible tracheal damage, whereas the attenuated mSD strain preserves ciliary integrity, allowing the cilia to beat normally and maintain their physiological function. Kyoto Encyclopedia of Genes and Genomes (KEGG) enrichment analysis further demonstrated that the two strains differed substantially in their ability to activate cytokine-storm–related pathways and innate immune signaling pathways (Fig 8C).

In the rH120 versus mH120 comparison, 2,073 DEGs were identified. The most prominently upregulated genes in rH120 infection were associated with antiviral and innate immune responses, such as RSAD2 and STAT1 (Fig 8D). GO enrichment analysis further indicated that the DEGs were mainly associated with immune recognition processes (Fig 8E), demonstrating marked differences in immune activation between the rH120 and mH120. This is consistent with the fact that rH120 is a commercial vaccine strain with a strong capacity to induce innate immune responses.

KEGG enrichment analysis further supported these observations (Fig 8F). Both the rSD/mSD and rH120/mH120 comparisons showed substantial differences in immune defense pathways, with the distinctions being even more pronounced in the rH120 groups. In addition to immune-related pathways, the rSD strain also activated multiple nucleotide and amino acid metabolism pathways, indicating a more complex pathological state and a greater extent of ciliary damage caused by the QX-type wild-type strain. Overall, the transcriptomic analyses indicate that rSD infection triggers more severe inflammation-related pathways than mSD, whereas the mammalian cell–adapted mH120 strain shows a substantially weaker induction of antiviral and innate immune responses.

### Infection with the BHK-adapted virus protects against WT rechallenge

**Fig 9:**
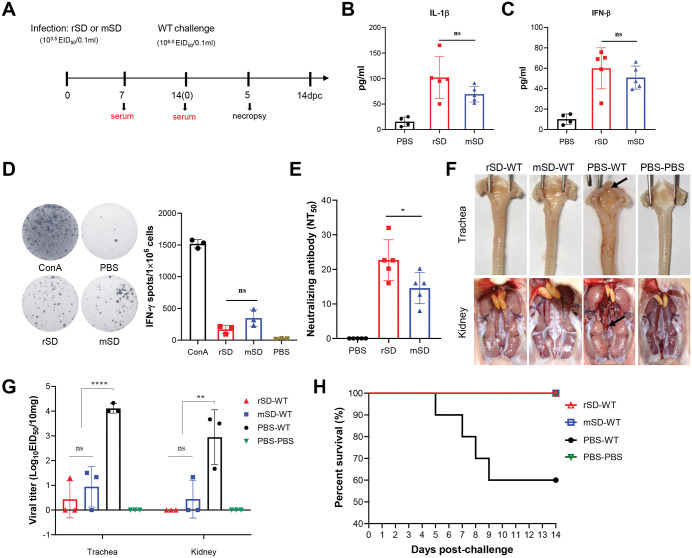
Protective efficacy of BHK-adapted IBV immunization. **(A)** Schematic of the experimental design: 1-day-old SPF chicks were inoculated with 10^3.5^ EID_50_ of rSD or mSD virus, followed by challenge with 10^6.0^ EID_50_ WT virus 14 days later. Serum samples were collected at 7 and 14 dpi, and necropsy was performed 5 days post-challenge. **(B)** Serum IL-1β levels at 7 days post-immunization. **(C)** Serum IFN-β levels at 7 days post-immunization. **(D)** IFN-γ ELISpot of splenocytes collected 14 days post-immunization. Cells were stimulated with T-cell epitope antigens, with ConA as the positive control. Spot numbers were quantified per 10^6^ cells. **(E)** Neutralizing antibody titers (NT₅₀). **(F)** Gross examination of trachea and kidney; black arrows indicate lesions. (G) Viral titers in trachea and kidney 5 days post-challenge. (H) Survival curves post-challenge (n = 10).

Chickens were immunized with 10^3.5^ EID_50_ of the BHK-adapted virus and challenged 14 days later with 10^6.0^ EID_50_ of WT (Fig 9A). To assess early innate immune responses, serum samples were collected at 7 days post-immunization (dpi) and IL-1β (Fig 9B) and IFN-β (Fig 9C) levels were measured. Although no statistically significant differences were observed among groups, the mSD-immunized group showed a trend toward reduction compared with rSD. At 14 dpi, three birds per group were randomly euthanized, and splenocytes were isolated to evaluate cellular immune responses by ELISpot assay. Both rSD and mSD induced detectable but relatively modest cellular immune responses (Fig 9D). Neutralization assays showed that rSD elicited approximately 1.5-fold higher neutralizing antibody titers than mSD (Fig 9E).

At 5 days post-challenge, marked tracheal mucus accumulation and evident urate deposition in the kidneys were observed exclusively in the unvaccinated challenge control group, whereas vaccinated groups showed no apparent gross lesions, comparable to the uninfected controls (Fig 9F). Viral titers in the trachea and kidney of both rSD- and mSD-immunized groups were significantly lower than those in the challenge control group, with no significant difference between rSD and mSD (Fig 9G). Importantly, mortality was 0% in both vaccinated groups, whereas the challenge control group exhibited 40% mortality (Fig 9H). Taken together, although mSD induced slightly lower humoral, innate, and cellular immune responses than the rSD group, it still provided effective protection against WT challenge showed by induction of cellular immunity, reduced tissue viral titers, absence of gross lesions, and full survival.

## Discussion

IBV is one of the major pathogens responsible for substantial economic losses in the global poultry industry [35]. Live-attenuated and inactivated vaccines remain the primary strategies for IBV prevention and control [36–38]. Currently, most IBV vaccines are produced using SPF embryonated chicken eggs [39]. However, this production system suffers from significant batch variability and biosafety concerns, and its sustainability is severely constrained by limited egg supply—particularly during outbreaks of avian influenza [40]. In contrast, mammalian continuous cell lines such as BHK and Vero cells possess advantages including rapid growth, batch-to-batch consistency, and suitability for large-scale culture, making them widely adopted for modern production of various viral vaccines [41]. Therefore, promoting IBV adaptation to mammalian cell lines is a critical prerequisite for replacing embryonated eggs with cell-based vaccine production platforms. Nevertheless, the strict host restriction of IBV has long prevented its replication in mammalian cells. Although the Beaudette strain, isolated in 1937, was adapted to Vero cells after serial passaging, it lost immunogenicity in chickens, limiting its application in vaccine development [42]. Moreover, Beaudette belongs to genotype GI-1, which differs substantially from the currently predominant GI-19 (QX-type) strains and thus fails to provide effective protection. Collectively, these facts highlight the importance of developing mammalian cell-adapted IBV platforms to enable scalable and secure vaccine production.

Previous studies have reported that the S2 subunit—particularly the S2′ furin cleavage motif (RRKR)—plays a critical role in determining the cell tropism of the IBV Beaudette strain [29]. Similarly, serial passaging of the H120 strain in Vero cells for 80 passages enabled its adaptation to mammalian cell lines [31]. However, these studies share a common limitation: they were all based on already cell-adapted IBV backbones (such as Beaudette) [43], rather than introducing the identified mutations into a wild-type background for functional verification. Moreover, they focused primarily on the spike protein as a necessary determinant of cell tropism, without establishing whether it is sufficient, and largely overlooked the contributions of other viral proteins during tropism expansion. Therefore, the identified key amino acid substitution, S (P687R), was introduced into the wild-type H120 backbone (rH120-S (P687R)); however, the recombinant rH120-S (P687R) virus failed to adapt to mammalian cells, a result consistent with our expectation [28,31]. Consistent with these findings, we attempted to introduce either the RRKR furin cleavage motif or the full-length Beaudette S gene into a prevalent field strain, but these modifications did not enable replication in mammalian cell lines. This result indicated that IBV adaptation to mammalian cells cannot be achieved through a few amino acid substitutions. More precisely, it is not solely determined by the S protein, as these changes could not accomplish a de novo gain of replication ability, but only the loss of an existing one. This clearly demonstrates that IBV cell adaptation involves not just the S protein, but also additional viral factors that collectively determine host range and replication competence.

Notably, previous studies showed that both Beaudette and H120 vaccine strains adapted to Vero cells acquired an S2′ furin cleavage site, and the introduction of this site into circulating field strains was reported to enhance viral virulence [32]. Therefore, we deliberately avoided Vero cells and instead selected the BHK-21 cell line as the platform for adaptation. However, passaging IBV in non-permissive cells often leads to viral loss. To overcome this, we adopted a strategy inspired by norovirus adaptation to human HeLa cells [44]—alternating passages between permissive (CEK) and non-permissive (BHK-21) cells. This approach leveraged the replication advantage of CEK cells while preventing the loss of viral infectivity in BHK-21 cells. Importantly, this alternating-passaging strategy requires a genetically diverse viral quasispecies population. To generate such diversity, we pre-passaged the wild-type IBV for 15 passages in embryonated chicken eggs and CEK cells before beginning adaptation. Under these conditions, we successfully obtained a BHK-21-adapted IBV strain.

Sequencing of the P20 population revealed numerous double peaks, indicating that the viral quasispecies remained highly complex, making it difficult to pinpoint the specific amino acid residues responsible for cell tropism. Therefore, we selected the 14 mutations that were consistently present across all plaque isolates, which were distributed among both structural and nonstructural proteins. Benefiting from our previously established high-efficiency reverse genetics system—based on the strong homologous recombination capacity of yeast—we were able to rapidly assemble full-length IBV genomes [45]. We first introduced the mutated S gene from rP_20_ into the rSD backbone, and as expected, only the recombinant virus carrying all 14 mutations was able to replicate efficiently in BHK-21 cells. However, acquisition of S mutations alone (rSD-S^*P20*^) was insufficient to confer rapid adaptation to BHK-21 cells. Therefore, the expansion of IBV tropism is not driven solely by the spike protein but rather by the coordinated contribution of both S and non-S proteins. Given that host range expansion is unlikely to result from a single mutation, we systematically reverted individual mutations to identify the key determinants of adaptation. This analysis revealed that mutations in nsp4 (T53I), E (E10G), and the S2 subunit collectively influenced IBV cell tropism. Among these, E (E10G) and S2 (D826Y, V852L) emerged early—between the wild-type (WT) and P_0_ generations—and nsp4 (T53I) also exhibited mixed peaks, during adaptation to BHK-21 cells, additional mutations emerged in the S2 subunit at residues 610, 864, and 868. These results demonstrate that IBV adaptation to mammalian cells is a gradual and cumulative process, in which mutations that enhance replication in embryonated eggs or CEK cells can also facilitate subsequent adaptation to mammalian cell lines. This underscores the importance of maintaining a diverse quasispecies population during early passages in eggs and CEK cells, which provides the genetic foundation for later adaptation. Thus, to be more specific, IBV adaptation to mammalian cells is primarily driven by spike mutations (Q610H, Q864L, Q868H); however, this process requires a prior avian-adapted genetic background acquired in eggs and chicken cells, including mutations in nsp4 (T53I), E (E10G), and S2 (D826Y, V852L). Importantly, this adaptation is driven by coordinated changes in both the spike protein and multiple non-S structural and non-structural proteins, as single S-protein mutations alone are insufficient to alter IBV cell tropism. Furthermore, we confirmed that these adaptive mutations also functioned in the H120 vaccine strain, as recombinant H120 carrying the same mutations produced CPE even at P_0_, demonstrating the broad applicability of these mutations. Animal challenge experiments showed that the BHK-21–adapted IBV exhibited attenuated pathogenicity in chickens, consistent with classical principles of viral attenuation—for instance, rinderpest virus (RPV) after 888 passages in rabbits or the rabbit-adapted classical swine fever virus (CSFV) vaccine strain [46,47]. In general, during adaptation to heterologous hosts, viruses gradually evolve to replicate efficiently in new hosts while becoming less virulent to their original hosts, Serial passage of a virulent wild-type virus in vitro often results in loss of virulence of the virus in an original animal host [48]. To further investigate the mechanisms underlying the attenuated pathogenicity, we performed transcriptomic analysis on tracheal tissues collected from chickens infected with the WT and the adapted mutant strains. The results revealed substantial differences in their host gene expression profiles. The SD strain strongly upregulated the pro-inflammatory cytokine IL8, whereas H120 infection induced higher expression of host antiviral and immune-related factors such as USP41 and STAT1. Differentially expressed genes were predominantly enriched in chemokine activity, consistent with enhanced inflammatory signaling. Moreover, KEGG enrichment analysis demonstrated that these DEGs were mainly associated with molecular functions related to the NOD-like receptor signaling pathway, cytokine–receptor interactions, and the Toll-like receptor signaling pathway, among others. These findings are in line with previous studies [49,50]. However, it should also be noted that attenuation may alter the virus-induced immune responses [51], potentially affecting its immunogenicity. This represents an important question that warrants further investigation in future studies.

In summary, this study, for the first time, used the prevalent QX-type IBV as a model to obtain a BHK-21 cell–adapted strain capable of efficient replication through serial passaging. We systematically dissected the key mutation combinations underlying the adaptation process, revealing that IBV adaptation to mammalian cells is not driven by a single amino-acid substitution but instead requires coordinated mutations across multiple genomic regions. This work highlights the complexity of IBV host-range determination and underscores the importance of epistatic interactions among nonstructural and structural proteins during cross-species adaptation. Our findings provide both theoretical and technical foundations for the rational design of broadly applicable vaccine strains and the development of cell culture–based IBV vaccines, offering a feasible strategy for transitioning IBV vaccine production from traditional embryonated eggs to modern cell-based manufacturing platforms.

## Materials and methods

### Animals and Ethics Statement

All specific-pathogen-free (SPF) chickens and SPF chicken embryos were purchased from Beijing Boehringer Ingelheim Vital Biotechnology Co., Ltd. (Beijing, China). The animal experiments were approved by the Animal Welfare and Ethical Censor Committee of China Agricultural University (Approval number: 2024–10062).

### Viruses and cells

The Mass-type vaccine strain H120 (GenBank accession No. MN548287) and the QX-type strain SD (GenBank accession No. KY421673) were preserved in our laboratory. Recombinant counterparts, rH120 and rSD, were rescued using a reverse genetics system. Baby hamster kidney (BHK-21) cells and chicken embryo fibroblast (DF-1) cells were obtained from ATCC. Primary chicken embryo kidney (CEK) cells were prepared from 18-day-old SPF chicken embryos. All cells were maintained in Dulbecco’s modified Eagle medium (DMEM; Gibco) supplemented with 10% fetal bovine serum (FBS), 100 U/mL penicillin, and 100 μg/mL streptomycin, and incubated at 37°C in a humidified atmosphere containing 5% CO₂.

### Alternating passage

The wild-type SD strain was inoculated into 9-day-old SPF embryonated chicken eggs, and allantoic fluid was collected at 36 h post-inoculation. The virus was serially passaged five times (E_5_). The E_5_ virus was then inoculated onto primary CEK cells and allowed to adsorb for 1 h at 37°C, followed by removal of unbound virus, three PBS washes, and the addition of maintenance medium containing 1% serum. After ten consecutive passages in CEK cells, a quasi-species population harboring multiple mutations was obtained and designated as P_0_.

The P_0_ was directly inoculated onto BHK-21 cells without dilution, allowed to adsorb for 1 h at 37°C, washed once with PBS, and then maintained in 1% serum–containing medium. Infected BHK-21 cultures from each passage (e.g., P_1_) were subjected to one to two freeze–thaw cycles before being inoculated into the next passage (P_2_). During the adaptation process, viruses from passages P2 and P5 were returned to CEK cells for amplification to recover the limited number of virions adsorbed to BHK-21 cells, thereby preventing the loss of IBV during alternating host–nonhost passaging. The amplified quasi-species were then used to resume passaging on BHK-21 cells, after which serial passaging was continued exclusively in BHK-21 cells until P_20_. The adaptation of IBV to DF1 cells was performed using the same alternating passage strategy as described for BHK cells.

### Construction of mutant viruses

Wild-type and mutant IBVs were rescued using a reverse genetics system as previously described [45,52]. Briefly, the full-length IBV genome was divided into 7–8 overlapping cDNA fragments, each sharing 30–50 bp of homologous sequences at their ends. A cytomegalovirus (CMV) promoter was fused to the 5′ untranslated region (UTR), the hepatitis delta virus (HDV) ribozyme and bovine growth hormone (BGH) polyadenylation signal were appended to the 3′ end. All fragments were seamlessly assembled in Saccharomyces cerevisiae by transformation-associated recombination (TAR). Yeast clones were lysed, and the assembled genome constructs were transformed into Escherichia coli to obtain sufficient plasmid DNA. For virus rescue, 2.5 μg of full-length genomic plasmid and 0.5 μg of a nucleocapsid (N) protein expression plasmid were diluted in 125 μL Opti-MEM medium (Thermo Fisher Scientific). In parallel, 6 μL Lipofectamine 2000 (Thermo Fisher Scientific) was diluted in 125 μL Opti-MEM and incubated for 5 min at room temperature. The DNA–lipid complexes were then mixed and incubated for 20 min before being added to BHK-21 cells at 80% confluency. After 4–6 h, the transfection medium was replaced with maintenance medium containing 1% FBS. At 48 h post-transfection, cells were subjected to three freeze–thaw cycles, and the supernatants were inoculated into 9-day-old SPF chicken embryos for viral amplification.

### Viral replication kinetics assay

As wild-type IBV does not replicate in BHK cells, replication kinetics of rescued viruses were uniformly quantified by viral genome copy number. Briefly, rescued viruses were inoculated onto BHK cells at a standardized input of 10^8^ genome copies. After 1 h of adsorption at 37°C, the inoculum was removed, and the cells were washed three times with sterile PBS before adding serum-free DMEM maintenance medium. At designated time points post-infection (12, 24, 48, 60, and 72 h), culture supernatants were collected. Viral titers were determined by endpoint dilution on BHK cells in 96-well plates, with cytopathic effects monitored for 4 days. 50% tissue culture infectious dose (TCID₅₀) was calculated using the Reed–Muench method.

### Plaque assay

BHK cells were seeded in 6-well plates and grown to approximately 90% confluence. Serial ten-fold dilutions of virus were inoculated onto the cells and incubated at 37°C for 1 h to allow adsorption. The inoculum was then removed, and the cells were overlaid with culture medium containing 1% low-melting-point agarose. After 4–5 days of incubation at 37°C, visible plaques appeared. The cell monolayers were fixed with 4% paraformaldehyde for 15 min, the agarose overlay was carefully removed, and the cells were stained with 0.1% Crystal Violet Staining Solution for 30–60 min at room temperature. Plaques were visualized and photographed.

### Indirect immunofluorescence assay (IFA)

At the indicated time points post-infection, cells were fixed with cold acetone/ethanol (3:2, v/v) for 20 min at −20°C and subsequently washed three times with PBST (PBS containing 0.1% Tween-20). Cells were then incubated overnight at 4°C with a monoclonal antibody against IBV S2 protein (1:5000, prepared in-lab) and a monoclonal antibody against IBV N protein (1:1000, HyTest, Turku, Finland). After three washes with PBST, cells were incubated with goat anti-mouse IgG secondary antibody conjugated with Alexa Fluor 555 (1:1000; Cell Signaling Technology, #8953) for 1 h at room temperature. Nuclei were counterstained with 4′,6-diamidino-2-phenylindole (DAPI; Cell Signaling Technology, #4083) for 10 min at room temperature. Fluorescence images were acquired using a Nikon microscope.

### Western blot

BHK cells infected with recombinant viruses were harvested at 24, 48, 60, and 72 h post-infection. Cells were washed three times with ice-cold PBS and lysed in RIPA buffer supplemented with protease inhibitors. Lysates were clarified by centrifugation at 12,000 × g for 5 min at 4°C, and the supernatants were mixed with loading buffer and boiled at 100°C for 10 min. Proteins were resolved by SDS–polyacrylamide gel electrophoresis (SDS-PAGE) and transferred onto PVDF membranes. Membranes were blocked with 5% skim milk for 1 h at room temperature, followed by three washes with TBST. Primary antibodies included a monoclonal antibody against the IBV S2 protein (1:5000 dilution, prepared in-lab) and a monoclonal antibody against the N protein (1:1000 dilution, HyTest, Turku, Finland), incubated overnight at 4°C. After washing, membranes were incubated with HRP-conjugated goat anti-mouse IgG secondary antibody (1:3000 dilution; CST, #7076) for 1 h at room temperature. Protein bands were visualized using ECL reagents (Beyotime Biotechnology, Shanghai, China).

### Measurement of total virus internalization

CEK and BHK cells were inoculated with a fixed viral copy number of 10^10^ copies per well. Cells were first incubated at 4°C for 1 h to allow virus adsorption, followed by five washes with PBS to remove unbound virions. Subsequently, cells were incubated at 37°C for 1 h to permit virus internalization. After incubation, residual extracellular virus particles were removed by washing with citrate-buffered PBS. Total cellular viral RNA was then extracted and quantified by RT-qPCR using the 2^-ΔΔCt^ relative quantification method. Viral genome copy numbers were determined by a SYBR Green-based qPCR assay targeting a highly conserved region of the 5′ untranslated region (Forward primer: 5′-GTTGGGCTACGTTCTCGC-3′; Reverse primer: 5′-AAGCCATGTTGTCACTGTCTAT-3′), and were calculated by interpolation from a standard curve previously established in our laboratory (Ct = −3.41·log₁₀x + 39.1, where x represents the viral copy number).

### Sequence alignment and conservation analysis

A total of 800 complete genome sequences of IBV representing major genotypes were downloaded from the NCBI GenBank database. The nsp, S, and E gene sequences were extracted using Geneious Prime. Multiple sequence alignments were performed with MAFFT, and the aligned sequences were visualized using WebLogo 3 - Create to generate sequence conservation plots for analysis of amino acid variation patterns. Amino acid conservation was assessed by sequence alignment, and residues with ≥95% identity among all analyzed IBV strains were defined as highly conserved.

### Animal experiments

The animal experiments were approved by the Animal Welfare and Ethical Censor Committee of China Agricultural University. One-day-old SPF chickens were randomly assigned to experimental and control groups (n = 20 per group; 10 for clinical observation and 10 for tissue sampling). Chickens in the experimental groups were inoculated with rSD or its BHK-adapted mutant mSD at a dose of 10^5.0^ EID_50_ per bird, rH120 or its adapted mutant mH120 was administered at a dose of 10^5.0^ TCID_50_ per bird. All inoculations were performed via the ocular and intranasal routes. Control chickens received PBS. On days 3, 5, and 7 post-inoculation, three birds per group were randomly selected for necropsy, and trachea and kidney were examined for gross pathological lesions. At the end of the 15-day observation period, all remaining birds were humanely euthanized by cervical dislocation to minimize suffering.

### Measurement of viral burden

Approximately 50 mg of tissue samples were homogenized in 500 μL of PBS. The homogenates were supplemented with antibiotics and incubated at 4°C overnight to remove bacterial contamination. Given the marked difference in replication between rSD and mSD in CEK cells, virus titers in tissue were determined by calculating the 50% egg infectious dose (EID₅₀) in specific-pathogen-free (SPF) embryonated chicken eggs. Tissue homogenates were serially diluted and inoculated into chicken embryos, and viral titers were calculated based on embryo curling lesions. CEK cells supported high-titer replication of both rH120 and mH120 viruses. Therefore, infectious virus titers in tissues collected from rH120- and mH120-infected chickens were quantified by determining the 50% tissue culture infectious dose (TCID_50_) in CEK cells.

### Histopathology

For histopathological analysis, tissue samples were fixed in 10% neutral-buffered formalin for more than 7 days, embedded in paraffin, sectioned, and stained with hematoxylin and eosin (H&E) for microscopic evaluation.

### Immunohistochemistry (IHC)

Paraffin-embedded tissue sections were deparaffinized, rehydrated, and immersed in distilled water, followed by incubation in 3% hydrogen peroxide solution for 15 min at room temperature to quench endogenous peroxidase activity. After washing with distilled water, sections were blocked with 1% bovine serum albumin (BSA) for 20 min at room temperature. The sections were then incubated overnight at 4°C with a mouse monoclonal antibody against the IBV N protein (1:1000; HyTest, Turku, Finland). Following PBS washes, the sections were incubated with an HRP-conjugated goat anti-mouse secondary antibody for 1 h at room temperature. Visualization was performed using 3,3′-diaminobenzidine chromogens (10 min, room temperature, in the dark), and sections were counterstained with hematoxylin.

### Analysis of RNA sequencing data

RNA sequencing of tracheal tissues collected at 5 days post-infection was performed by Benagen Technology Co., Ltd. (Wuhan, China). Raw FASTQ reads were quality-controlled and filtered using fastp. Clean reads were aligned to the chicken reference genome. Differential expression analysis was performed with DESeq2 in R using raw counts as input. P-values were adjusted for multiple testing using the Benjamini–Hochberg procedure (FDR). Differentially expressed genes (DEGs) were defined as those with FDR < 0.05 and |log2 fold change| > 1.0.

### Challenge experiment

One-day-old chicks were inoculated with recombinant rSD or mSD viruses of 10^3.5^ EID_50_ per 0.1 mL. Chickens in the negative control group received 100 μL of PBS. Each group consisted of 20 chicks. At 7 days post infection, blood samples were randomly collected to assess innate immune responses. At 14 dpi, serum samples were collected for the determination of neutralizing antibody titers. At 14 dpi, three chickens from each group were euthanized, and splenic lymphocytes were isolated. T cell responses were evaluated by measuring IFN-γ secretion using an ELISpot assay kit (Mabtech, Sweden). At 14 dpi, the remaining chickens were challenged with the WT virus at a dose of 10^6.0^ EID_50_ per chicken. An additional challenge control group (A total of 13 chickens were included in each group, of which 10 were monitored for mortality) was included and inoculated with the same dose of WT virus. Following challenge, clinical signs were monitored every day. At 5 days post challenge (5 dpc), three chickens from each group were randomly euthanized for necropsy. Trachea and kidney tissues were collected for gross lesion examination and viral load determination.

### Cytokine and chemokine protein measurements

At 7 days post immunization, serum samples were randomly collected from five chickens. The concentrations of IL-1β were determined using a commercial ELISA kit (Cloud-Clone Corp., Wuhan, China), while IFN-β levels were measured using an ELISA kit (Nanjing SenBeijia Biological Technology Co, Ltd, China).

### Statistical analysis

The data were expressed as mean ± SEM. Statistical analyses were performed using two-way analysis of variance (ANOVA) with GraphPad Prism 8.0, and differences were considered statistically significant at P < 0.05.

## Supporting information

S1 Fig (A) BHK-21 cells were infected at an MOI of 0.1, and supernatants collected at the indicated time points were subjected to endpoint titration on CEK cells. (B) Comparison of viral titers of WT, P0, P10, and P20 in CEK cells. (C) Growth kinetics of WT, P0, P10, and P20 in BHK-21 cells, determined by viral genome copy number quantification. (D) Schematic diagram of alternating passages in DF1 cells. This image was created with https://BioRender.com/hz06xz2. (E) Viral titers of the adapted virus in DF1 cells following alternating passages. (F) IFA analysis of BHK-21–adapted virus infection in different mammalian cell lines.
(TIF)

S2 Fig Sequencing results of the rescued rH120-based recombinant virus carrying the nsp4 (T53I), E (E10G), and S2 subunit mutations after eight passages (P8). (TIF)

S3 Fig Effects of camostat and E64d concentrations on cell viability. (A) CEK cells. (B) BHK-21 cells.
(TIF)

S1 Data The datasheet includes all raw data related to viral replication, virus titration, and mutation analysis. (XLSX)
