# Supplementary figures and images for: Accumulation of mutations in nsp4, E, and the S2 subunit underlies mammalian cell tropism expansion and virulence attenuation of avian coronavirus
Source: PLoS Pathog. 2026 Apr 9;22(4):e1014147. doi: 10.1371/journal.ppat.1014147 (PMC13086431; doi:10.1371/journal.ppat.1014147)

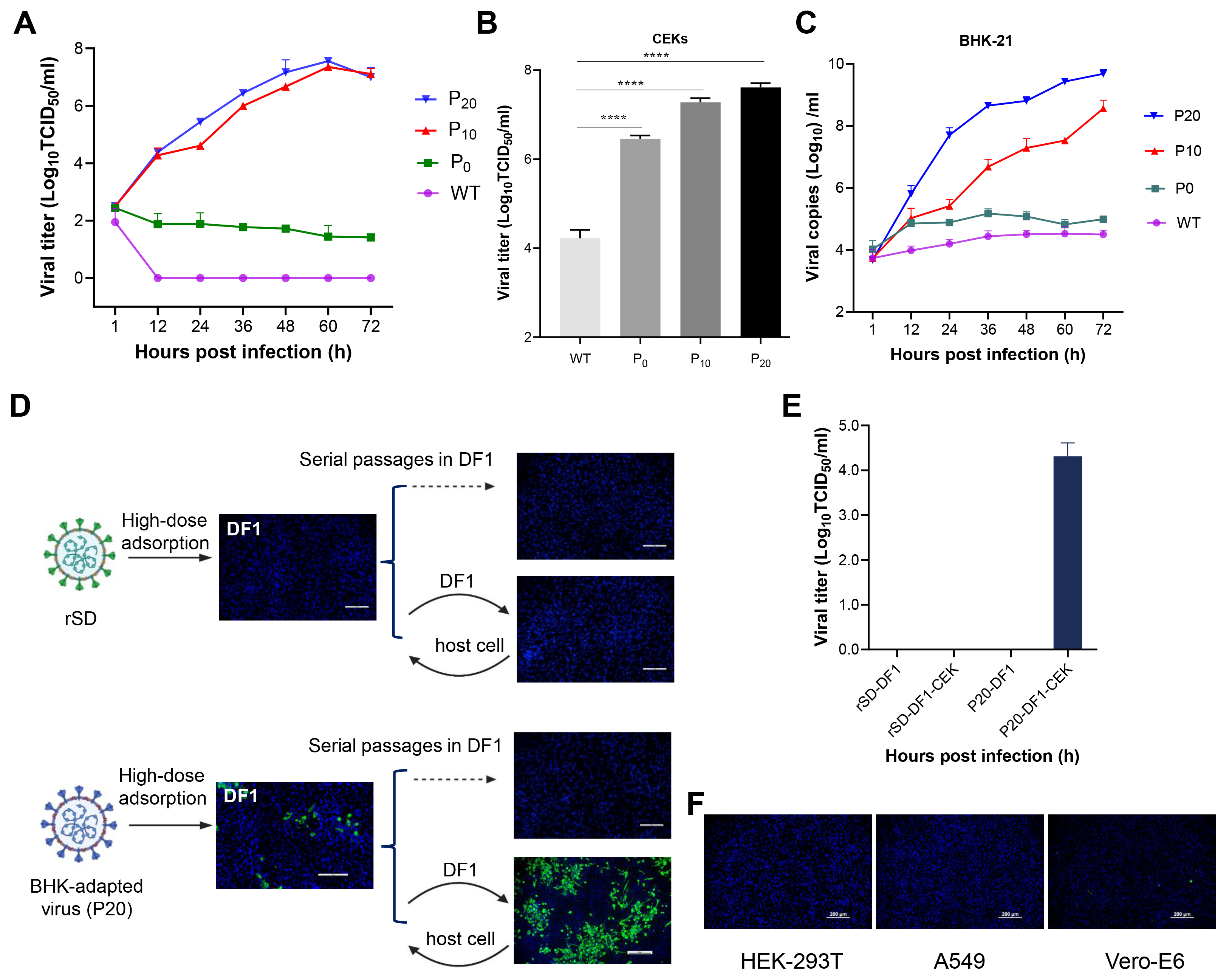

Supplement: S1 Fig — (B) Comparison of viral titers of WT, P0, P10, and P20 in CEK cells. (C) Growth kinetics of WT, P0, P10, and P20 in BHK-21 cells, determined by viral genome copy number quantification. (D) Schematic diagram of alternating passages in DF1 cells. This image was created with https://BioRender.com/hz06xz2. (E) Viral titers of the adapted virus in DF1 cells following alternating passages. (F) IFA analysis of BHK-21–adapted virus infection in different mammalian cell lines. (TIF) [file ppat.1014147.s001.tif]

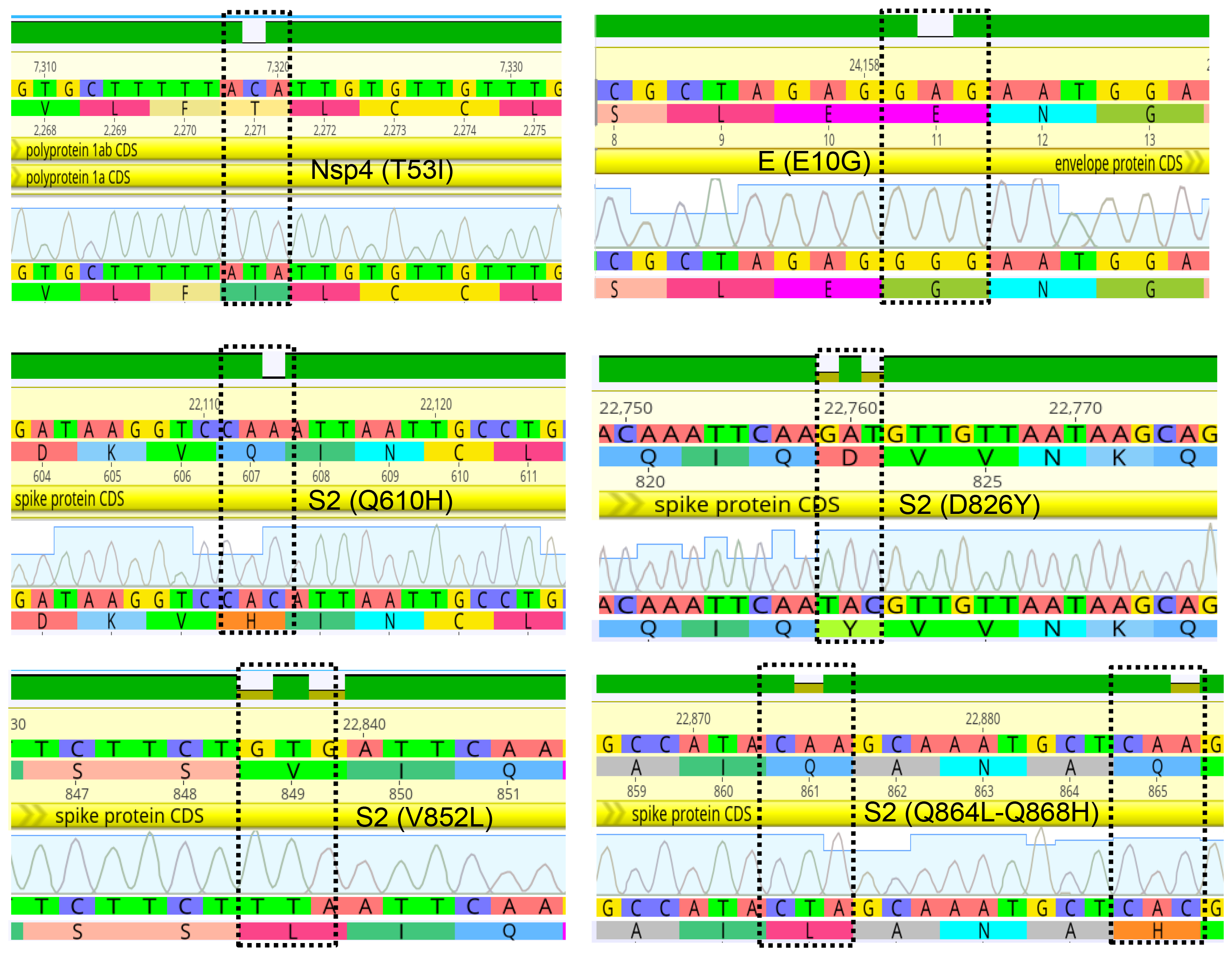

Supplement: S2 Fig — (TIF) [file ppat.1014147.s002.tif]

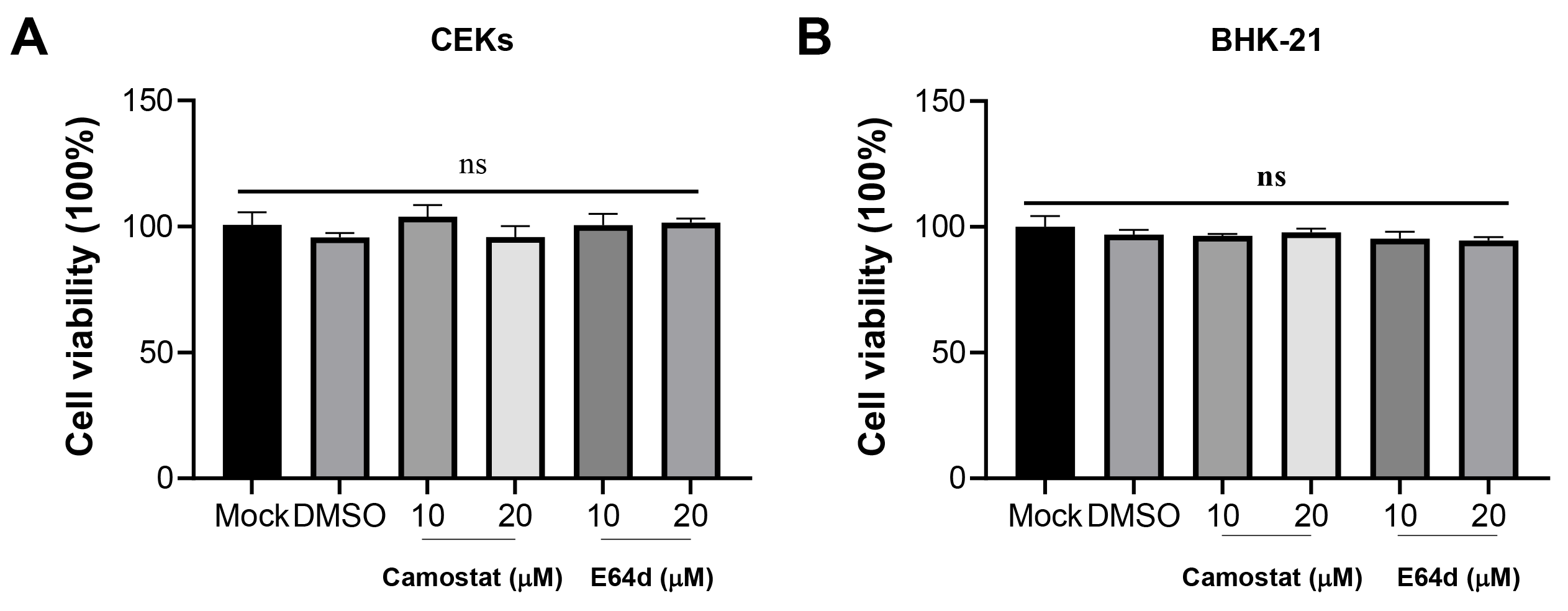

Supplement: S3 Fig — (A) CEK cells. (B) BHK-21 cells. (TIF) [file ppat.1014147.s003.tif]
